# Predicting intercellular communication based on metabolite-related ligand-receptor interactions with MRCLinkdb

**DOI:** 10.1186/s12915-024-01950-w

**Published:** 2024-07-08

**Authors:** Yuncong Zhang, Yu Yang, Liping Ren, Meixiao Zhan, Taoping Sun, Quan Zou, Yang Zhang

**Affiliations:** 1 Guangdong Provincial Key Laboratory of Tumor Interventional Diagnosis and Treatment, Zhuhai Institute of Translational Medicine, Zhuhai People’s Hospital (Zhuhai Clinical Medical College of Jinan University), Jinan University, Zhuhai, Guangdong China; 2 Innovative Institute of Chinese Medicine and Pharmacy, Academy for Interdiscipline, Chengdu University of Traditional Chinese Medicine, Chengdu, China; 3 School of Healthcare Technology, Chengdu Neusoft University, Chengdu, China; 4 Institute of Fundamental and Frontier Sciences, University of Electronic Science and Technology of China, Chengdu, China

**Keywords:** Metabolite, Ligand-receptor interaction, Cell-cell communication, Single cell RNA sequencing, Spatial transcriptomics

## Abstract

**Background:**

Metabolite-associated cell communications play critical roles in maintaining human biological function. However, most existing tools and resources focus only on ligand-receptor interaction pairs where both partners are proteinaceous, neglecting other non-protein molecules. To address this gap, we introduce the MRCLinkdb database and algorithm, which aggregates and organizes data related to non-protein L-R interactions in cell-cell communication, providing a valuable resource for predicting intercellular communication based on metabolite-related ligand-receptor interactions.

**Results:**

Here, we manually curated the metabolite-ligand-receptor (ML-R) interactions from the literature and known databases, ultimately collecting over 790 human and 670 mouse ML-R interactions. Additionally, we compiled information on over 1900 enzymes and 260 transporter entries associated with these metabolites. We developed Metabolite-Receptor based Cell Link Database (MRCLinkdb) to store these ML-R interactions data. Meanwhile, the platform also offers extensive information for presenting ML-R interactions, including fundamental metabolite information and the overall expression landscape of metabolite-associated gene sets (such as receptor, enzymes, and transporter proteins) based on single-cell transcriptomics sequencing (covering 35 human and 26 mouse tissues, 52 human and 44 mouse cell types) and bulk RNA-seq/microarray data (encompassing 62 human and 39 mouse tissues). Furthermore, MRCLinkdb introduces a web server dedicated to the analysis of intercellular communication based on ML-R interactions. MRCLinkdb is freely available at https://www.cellknowledge.com.cn/mrclinkdb/.

**Conclusions:**

In addition to supplementing ligand-receptor databases, MRCLinkdb may provide new perspectives for decoding the intercellular communication and advancing related prediction tools based on ML-R interactions.

## Background

Cellular communication forms the foundation of intercellular connections within biological systems, transcending diverse biological forms and tissue structures [1, 2]. In multicellular organisms, intercellular communication plays a pivotal role in various physiological processes, such as immune responses, neural transmission, and developmental regulation [2–4]. Immune cells utilize intercellular communication to identify and combat invading pathogens, maintaining immune homeostasis within the organism [5]. Neuronal cells achieve rapid signal transmission through the release and reception of neurotransmitters [6]. During the organism’s developmental journey, intercellular communication orchestrates cell differentiation, tissue formation, and organ development [7]. Furthermore, intervening in intercellular communication stands as a strategic approach, allowing for the modulation of aberrant signal transduction to restore normal physiological function [8, 9]. Therefore, a comprehensive understanding of intercellular communication mechanisms can offer a theoretical foundation for designing novel drugs and therapeutic approaches [10].

In recent years, propelled by the rapid advancement of single-cell sequencing and spatial transcriptomics technologies, the systematic exploration of intercellular communication within tissues and microenvironments has emerged as a focal point in current research [11–15]. Over the past 3 years, more than 80 algorithms and data resources pertaining to intercellular communication have been developed (https://www.cellknowledge.com.cn/mrclinkdb/LitetatureCCI.html). These include methods based on the expression of cell-to-cell ligand-receptor (L-R) interactions (such as CellChat [16], CellPhoneDB [17], SingleCellSignalR [18], NATMI [19], ICELLNET [20], etc.), methods based on downstream intracellular signaling network of L-R interactions (CellCall [21], CCCExplorer [22], SoptSC, NicheNet [23], CytoTalk [24], scMLnet [25], etc.), and methods incorporating spatial transcriptomics data (Cell2Cell [26], SpaOTsc [27], SVCA [28], stLearn [29], COMMOT [30], etc.).

**Fig. 1:**
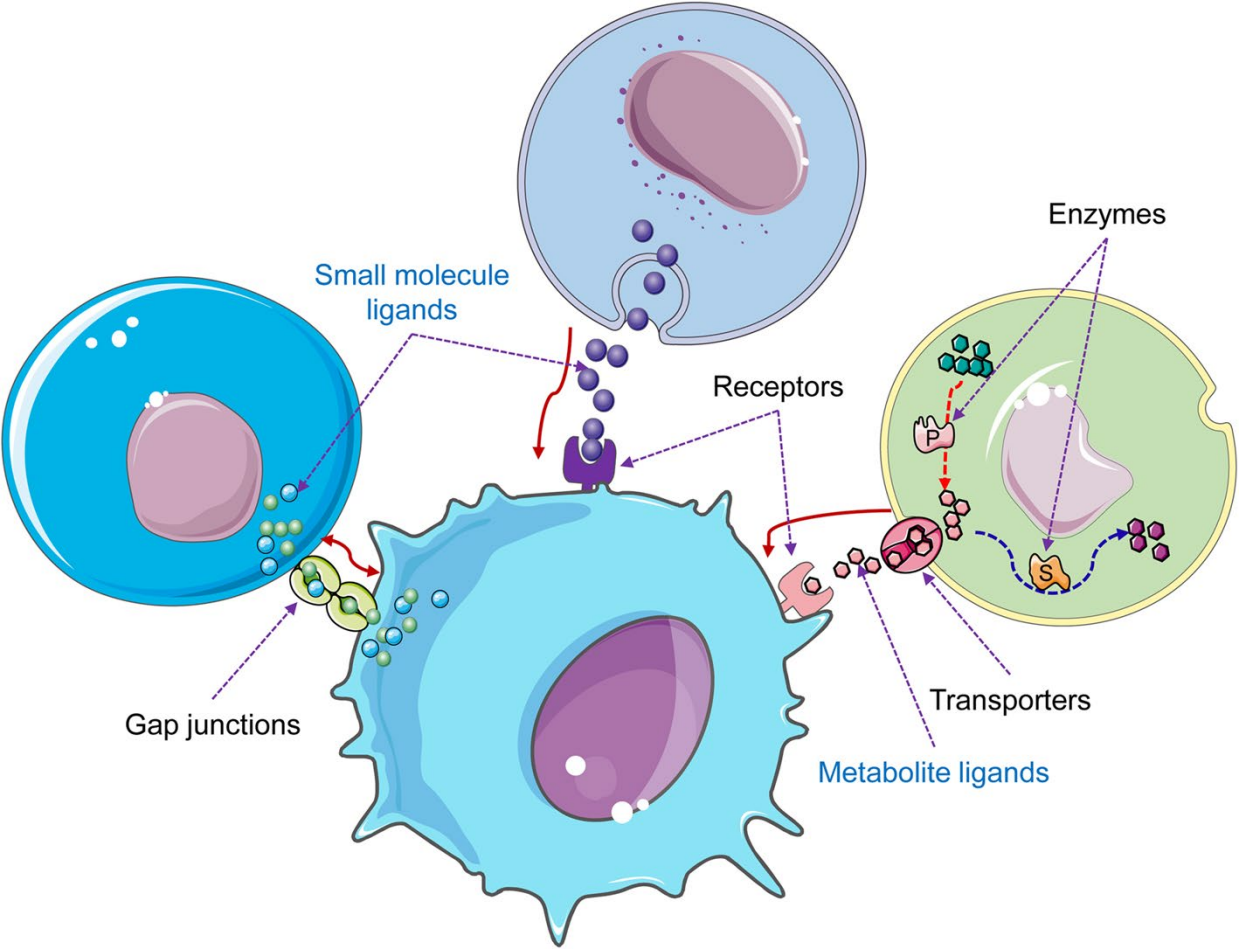
Schematic diagram of ML-R interaction-based intercellular communication

The aforementioned cell-cell communication (CCC) prediction methods based on single-cell sequencing all rely on prior knowledge of L-R interactions [31]. Therefore, apart from the L-R interaction resources such as DLRP [32], IUPHAR/BPS [33], KEGG [34], and HPMR [35] existing before the single-cell sequencing era, specialized resources targeting CCC research have also emerged, such as Cellinker [36], connectomeDB2020 [17], CellTalkDB [37], OmniPath [38], and PlantPhoneDB [39]. Meanwhile, there are existing databases that provide cell-cell communication analysis results for individual datasets, which can be directly accessed and explored by researchers. These include CellCommuNet [40], ABC portal [41], AgeAnno [42], HTCA [43], and SPEED [44]. However, most current methods and platforms primarily focus on those cell communications and L-R pairs in which both partners are proteinaceous. Yet, the material foundation of intercellular communication extends beyond proteins and includes numerous non-protein endogenous ligands such as carbohydrates, lipids, inorganic compounds, metal ions, and nucleic acid ligands (Fig. 1). Among these, metabolites are classical non-protein ligands that play significant roles in cell-to-cell interactions [45–48]. For instance, the interaction between tumor-derived histamine and macrophage histamine receptor H1 (HRH1) can lead to T cell dysfunction [49]. Neurotransmitters, typically non-peptide small molecules such as glutamate and gamma-aminobutyric acid (GABA), are crucial for communication between neurons, influencing various physiological processes and behaviors. Glutamate and GABA not only facilitate signal transmission between neurons but also play important roles in neurons-microglia interactions [50]. Additionally, cancer cells often export lactate, which induces a local inflammatory response in the tumor environment, ultimately promoting tumor cell growth and metastasis [51–53].

Presently, a limited number of studies have initiated the development of intercellular communication prediction methods based on non-protein L-R interactions [54]. Zheng et al. introduced the MEBOCOST algorithm, which leverages the expression of various enzymes related to metabolites to predict intercellular communication involving diverse metabolites [54]. Zhao et al. devised NeuronChat [55], a tool based on the expression of enzymes and transport proteins linked to neurotransmitters (including many non-protein ligands), enabling the inference of communication between neurons. In addition to extensively cataloging L-R protein-protein interactions (PPIs), our previous work, Cellinker [36], has also compiled over 400 small molecule-related L-R interaction data. These endeavors notably expand the application scope of intercellular communication prediction tools. Nonetheless, there remains a lack of a dedicated resource that aggregates and organizes data related to non-protein L-R interactions in CCC.

**Fig. 2:**
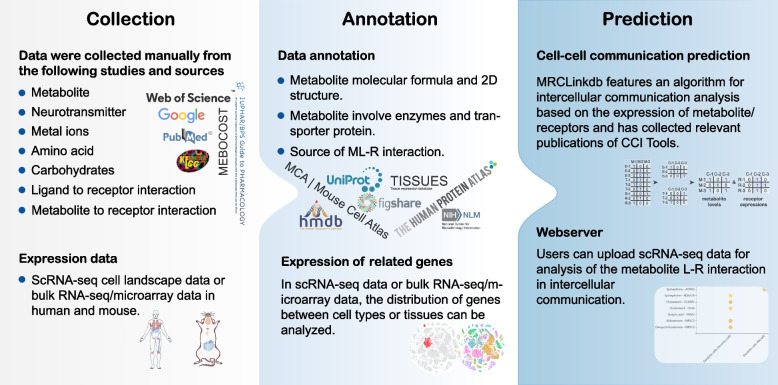
Construction and content of MRCLinkdb

In this study, we developed MRCLinkdb, a resource of metabolite-ligand-receptor (ML-R) interactions for intercellular communication analysis (Fig. 2). The current version of MRCLinkdb documents more than 790 human and 670 mouse ML-R interactions. Additionally, the platform has compiled information on over 1900 enzyme entries and 260 transporter entries associated with these metabolites. The platform offers a user-friendly interface and a wealth of information, facilitating the browsing and presentation of ML-R interactions, and introduces a new webserver dedicated to the analysis of the ML-R interaction-based intercellular communication. MRCLinkdb is free available at https://www.cellknowledge.com.cn/mrclinkdb/.

## Results

### Statistics of ML-R interactions

**Fig. 3:**
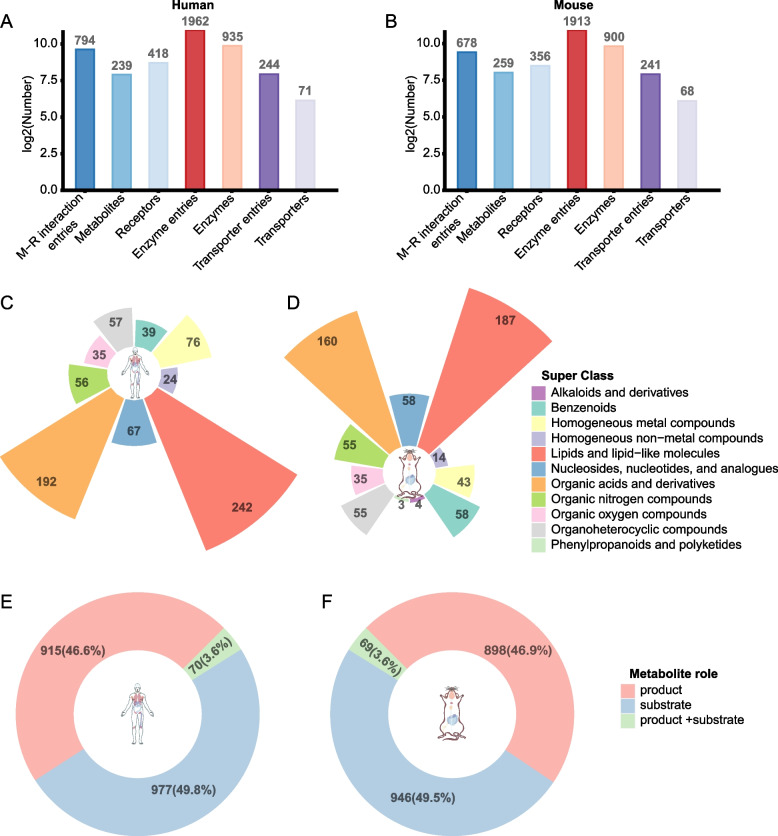
Statistical data for MRCLinkdb. **A** Statistics for human ML-R interactions, enzymes, and transporter proteins. **B** Statistics for mouse ML-R interactions, enzymes, and transporter proteins. **C** Category distributions of metabolites related to human ML-R interaction. **D** Category distributions of metabolites related to mouse ML-R interaction. **E** Role of human enzymes in metabolite-related reactions. **F** Role of mouse enzymes in metabolite-related reactions

The MRCLinkdb platform offers a comprehensive collection of ML-R interactions. For humans, the platform hosts 794 ML-R interactions, referencing 239 metabolites and 418 receptor proteins or complexes (Fig. 3A). Furthermore, it documents 1962 enzyme entries (related to 935 unique enzymes) and 244 transporter entries (related to 71 unique transporters). For mouse, the platform hosts 678 ML-R interactions, referencing 259 metabolites and 356 receptor proteins or complexes (Fig. 3B). It also documents 1913 enzyme entries (related to 900 unique enzymes) and 241 transporter entries (related to 68 unique transporters).

Categorically, human metabolites are distributed across nine types, including 39 benzenoids, 76 homogeneous metal compounds, 24 homogeneous non-metal compounds, 242 lipid-type interactions, 67 nucleosides, nucleotides, and analogues, 192 organic acids and derivatives, 56 organic nitrogen compounds, 35 organic oxygen compounds, and 57 organoheterocyclic compounds (Fig. 3C). Mouse metabolites are categorized into 11 classes, including 4 alkaloids and derivatives, 58 benzenoids, 43 homogeneous metal compounds, 14 homogeneous non-metal compounds, 187 lipids, 58 nucleosides, nucleotides, and analogues, 160 organic acids and derivatives, 55 organic nitrogen compounds, 35 organic oxygen compounds, 55 organoheterocyclic compounds, and 3 phenylpropanoids and polyketides (Fig. 3D).

Evaluating the role of enzymes, 915 human enzyme entries (46.6%) are identified as producers of their associated metabolites. Conversely, 977 entries (49.8%) participate in the consumption of the metabolite, with an additional 70 entries (3.6%) having the dual capacity to both generate and use the corresponding metabolite (Fig. 3E). A similar pattern is seen in mouse, with 898 enzyme entries (46.9%) for production, 946 entries (49.5%) for consumption, and 69 entries (3.6%) capable of both roles (Fig. 3F).

### MRCLinkdb interface overview

The MRCLinkdb platform offers an intuitive interface composed of six functional modules designed to access and explore ML-R interaction data. These modules include “search”, “browse”, “webserver”, “submit”, “download & API”, and a “list of CCI tools”. The “browse” module allows easy navigation of all ML-R interactions, with a “more” option in each row granting detailed insights into selected entries. It offers extensive data annotations and strives to present the data as visually as possible. The “download & API” section ensures comprehensive data accessibility, while the “list of CCI tools” presents a curated collection of CCC prediction research from the past 3 years. Users are also encouraged to contribute missing ML-R interaction data through the “submit” page.

### Data querying and result presentation

**Fig. 4:**
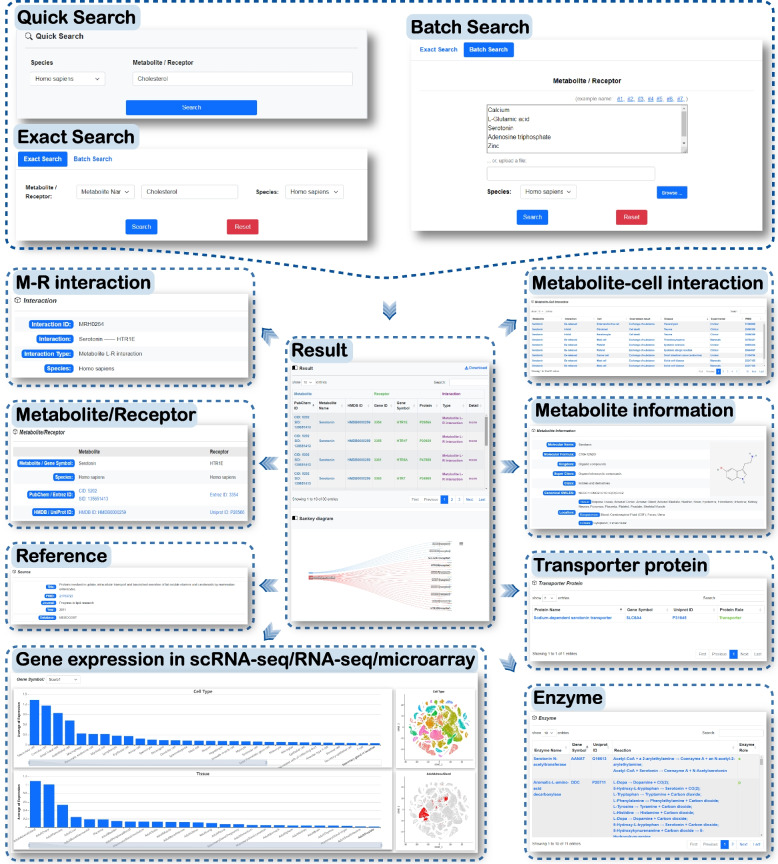
The Search page, Result page, and Detail page of MRCLinkdb

To facilitate user access and browsing of ML-R interaction data, MRCLinkdb has designed an elaborate search and browsing interface. The overall design and layout of the website are illustrated in Fig. 4. If users wish to search for specific metabolites and/or proteins, they can utilize the “search” interface. MRCLinkdb offers two distinct search methods: “Exact Search” and “Batch Search”. For these searches, users are required to input or upload details such as the metabolite name, HMDB ID, PubChem CID, PubChem SID, gene symbol, Entrez ID, or UniProt ID of the metabolites/receptors (Fig. 4). The search outcomes are displayed in a tabular format accompanied by an embedded Sankey plot. For more in-depth information regarding specific ML-R interactions, users can navigate to the Detail page by clicking on “more”. This detailed page provides extensive data, including (1) basic information about M-R interactions, such as species, metabolite name, HMDB ID, PubChem CID, PubChem SID, gene symbol, Entrez ID, UniProt ID, and source; (2) fundamental details of the metabolite, like its 2D structure, molecular formula, kingdom, class, canonical SMILES, and the associated enzymes and transporter proteins of metabolic reactions; (3) source and destination of metabolites, such as metabolite tissue, biospecimen, and cellular location information, and metabolite-cell interaction information; and (4) expression data for the receptor, enzymes, and transporter proteins based on scRNA-seq (covering tissues and cell types) and bulk RNA-seq/microarray data (encompassing tissues). Furthermore, by hovering over the bar chart of the scRNA-seq, users can highlight and view the distribution of tissues/cell types they are interested in. The right panel will then display the distribution of these tissues/cell types, helping users in understanding the expression distributions of receptors, enzymes, and transporter proteins more clearly.

### Online tool for analyzing ML-R interaction-based intercellular communication

**Fig. 5:**
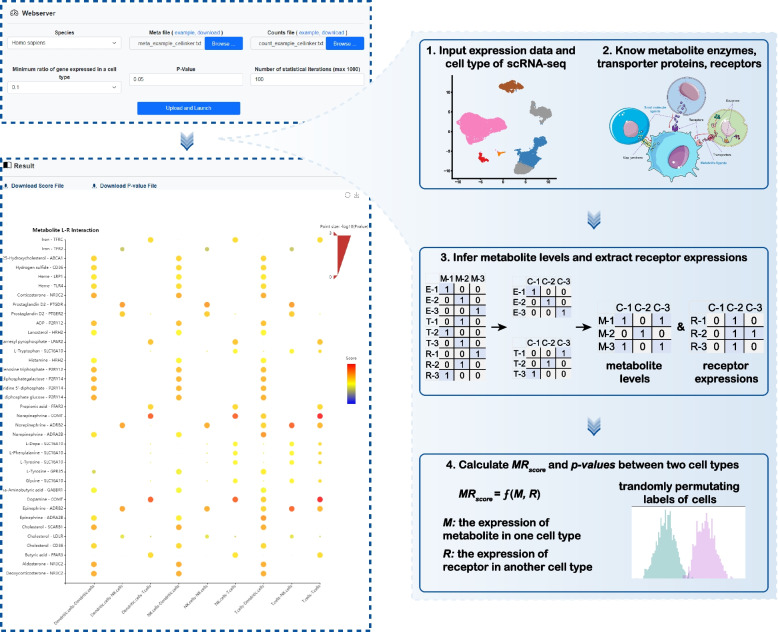
Webserver and algorithm for ML-R interaction-based intercellular communication analysis

MRCLinkdb introduces a dedicated webserver to facilitate analysis of intercellular communication via metabolite and receptor interactions, leveraging scRNA-seq data (Fig. 5). Users are required to upload an expression matrix file (either counts or TPM). In this matrix, rows correspond to gene symbols while columns represent individual cells. An accompanying META file should be provided, detailing each cell’s name and type. The platform offers a customizable experience. Users can designate a threshold “*N*”, thereby filtering out enzymes, transporter proteins, or receptors expressed in fewer than “*N*%” of cells within a specific cell type. There is also flexibility to define the *p-value* threshold and stipulate the number of iterations for the permutation test. Upon analysis completion, outcomes, including *MR*_score_ and *p*-values, are displayed as a visually intuitive bubble plot on the result page. Additionally, users have the provision to download the data for offline perusal (Fig. 4).

## Discussion

MRCLinkdb functions as a comprehensive platform for analyzing ML-R interactions in intercellular communication. However, it still presents certain limitations. Firstly, MRCLinkdb provides detailed annotations for ML-R interactions, yet it currently lacks the capacity to offer insights into the specific biological processes or pathways in which these interactions are involved. A more nuanced understanding of these processes is crucial for comprehending the broader implications of ML-R interactions within distinct biological contexts. Meanwhile, the resource predominantly focuses on enzymes directly associated with metabolites but does not encompass vital enzyme-related chemical reaction constants, such as Km, Ks, and Kcat. These constants are indispensable for precisely estimating metabolite concentrations. Additionally, the scope of MRCLinkdb is primarily limited to data related to metabolite-linked transport proteins. However, the field of metabolism encompasses a wide array of transport mechanisms beyond transporter-mediated pathways. Mechanisms such as exosome and vesicle trafficking, along with their associated molecules, remain absent from the resource. Recognizing these diverse aspects of metabolism is essential for a more comprehensive understanding of intercellular communication.

MRCLinkdb’s webserver tool employs methods from MEBOCOST and NeuronChat, using enzyme RNA expression levels to estimate the extracellular concentration of related metabolites and thus infer intercellular communication. However, this approach has limitations: (1) the relationship between enzyme mRNA expression levels and protein content is nonlinear, potentially introducing bias when inferring metabolite levels; (2) metabolite concentrations are also influenced by upstream substrates, downstream by-products, and environmental factors such as temperature and pH, which are not fully captured by transcription data alone; (3) high expression levels of metabolites and receptors do not necessarily indicate actual intercellular communication, adding uncertainty to predictions based solely on ligand-receptor levels. While single-cell metabolomics or proteomics data would provide more reliable insights, these techniques currently lag behind transcriptomics in molecular coverage and throughput. Therefore, single-cell and spatial transcriptomics data remain the primary resources for these inferences. Moreover, few algorithms are specifically designed to infer metabolite-receptor interactions. Despite potential high false positive rates, existing algorithms still offer valuable preliminary insights into intercellular communication mechanisms. In conclusion, although using enzyme and transporter protein expression levels to infer metabolite-related intercellular communication has limitations, it remains a useful approach given current technological constraints. We look forward to advancements in biotechnology that will improve the accuracy and interpretability of these predictions.

In the evolving landscape of research concerning ML-R interactions, we are firmly committed to maintaining the integrity of our platform by conducting regular updates. Our primary objective is to ensure data accuracy and comprehensiveness as new insights continue to emerge. One of our foremost goals is to broaden the taxonomic scope of our platform, encompassing a wider array of model organisms and systems pertinent to various diseases. This expansion will facilitate a more comprehensive understanding of ML-R interactions in diverse biological contexts. Moreover, our future endeavors will focus on incorporating additional functional data. We intend to highlight the dynamic changes in ML-R interactions across different disease states and elucidate the intricate relationships between ML-R interactions and cellular fate determinations. This expansion will provide a richer set of annotations for ML-R data. To further enrich the dataset, we plan to integrate detailed metabolite-related information. This will include enzyme reaction constants and critical binding constants that serve as bridges between metabolites and receptors. This addition will significantly enhance the depth and breadth of our ML-R data annotations. At last, leveraging these enhanced datasets, we will proceed to refine ML-R prediction methodologies and further enhancing the functionalities of our online server.

## Conclusions

MRCLinkdb represents an integrated platform that combines ML-R interaction data collection, curation, and intercellular communication analysis. It catalogs over 790 human and 670 mouse ML-R interactions and compiles information on more than 1900 enzyme entries and 260 transporter protein entries associated with these metabolites. The platform offers a user-friendly interface and comprehensive information, facilitating the exploration and visualization of ML-R interactions. Additionally, it introduces a dedicated web server for intercellular communication analysis based on ML-R interactions. In summary, we believe that this platform possesses invaluable potential to expedite the unraveling of intercellular communication intricacies and propel the progress of algorithms related to scRNA-seq and spatial transcriptomics data, especially those geared towards predicting CCC based on ML-R interactions.

## Methods

### Collection of ML-R interaction

The ML-R interaction data were manually curated from literatures and databases (Fig. 2). For curation, we search for literatures with the following keywords in Web of Science, PubMed and Google: “metabolite”, “secreted metabolite”, “metabolite cell–cell communication”, “extracellular metabolite”, “metal ions”, “neurotransmitter”, “amino acid”, “carbohydrates”, “ligand to receptor interaction”, “metabolite to receptor interaction”. The retrieved publications were then subjected to a preliminary check by expert curators to eliminate false-positive papers. Only experimentally supported ML-R interactions were included in the MRCLinkdb platform. Meanwhile, we also collected the ML-R interactions from three other known resources, including IUPHAR/BPS [33], KEGG [56], and MEBOCOST [54]. The location information of metabolites in tissues, biological specimens, and cellular was collected from HMDB. And the metabolite-cell interaction pairs were collected from MACC [57]. In addition, in order to better assist users in understanding the latest developments and dynamics in CCC prediction research, we have compiled a list of prediction tools and resources from the past 3 years. This compilation, available on our platform (https://www.cellknowledge.com.cn/mrclinkdb/LitetatureCCI.html), includes over 80 tools and databases and will be periodically updated to reflect the latest advancements.

### Data annotation

To enhance the usability of the data within the platform, we have performed rigorous annotation of ML-R interaction data, referencing several authoritative resources (Fig. 2). For metabolite details, annotation such as the molecular name, HMDB ID, CID, SID, kingdom, super class, class, molecular formula, canonical SMILES, and 2D structure of metabolite were sourced from NCBI PubChem database [58]. The metabolic reactions, enzymes, and transporter proteins associated with the metabolite were sourced the Human Metabolome Database (HMDB) [59]. The relationship between metabolites and enzymes primarily arises from the chemical reactions involving small molecules catalyzed by enzymes. For gene details, we referenced the NCBI Gene database [60] to acquire the gene name and entrez ID. Protein-related details were obtained from UniProt database [61]. It is worth noting that while the HMDB exclusively offers information on human-related enzymes and transporter proteins, we bridged this gap for mouse enzymes and transporter proteins using gene homology mapping. Orthology data for enzymes and transporter proteins was gathered from HomoloGene [60].

### scRNA-seq and bulk RNA-seq data collection and processing

Human and mouse single cell atlas were collected from Mouse Cell Atlas (MCA) version 1.0 [62] and version 1.1 [63]. The human cell landscape contains 344,000 cells, consisting of 52 cell types across 35 tissues. The mouse cell landscape contains 174,455 cells, consisting of 44 cell types and 26 tissues. The bulk RNA-seq/microarray data for gene expression across different tissues in human and mouse were collected from the Human Protein Atlas (HPA) project (62 human tissues) [64] and the TISSUES 2.0 database (39 mouse tissues) [65], respectively.

For further processing, we procured scRNA-seq raw data and any supplementary data provided by the authors. The raw data of human was analyzed by the Seurat R package (version = 4.0.5) [66]. Besides otherwise stated, all parameters use the default values. Cells registering fewer than 100 detected genes were excluded to maintain data quality. The expression matrix, post-filtering, was normalized using Seurat’s NormalizeData function (with a scale.factor set at 10,000). Leveraging the FindVariableFeatures function, 3000 highly variable genes were identified, forming the basis for subsequent principal component analysis. The tissue and cell type distributions were visualized through t-Distributed Stochastic Neighbor Embedding (tSNE) via the RunTSNE function (dims spanning 1:50). The average gene expression was determined for each tissue and cell type. A parallel analysis approach was adopted for the mouse scRNA-seq dataset.

### Inferring ML-R interaction-based cell-cell communication

MRCLinkdb employed a webserver to infer cell communication based on metabolites and their receptor proteins. The methodological approach was inspired by the MEBOCOST [54] and NeuronChat [55] algorithms. By contrast, MRCLinkdb’s adoption of the Euclidean norm in its algorithm for inferring intercellular communication scores improve sensitivity, interpretability, and robustness. The Euclidean norm ensures balanced consideration of metabolite concentration and receptor expression levels, enhancing sensitivity to their variations and allowing for additive effects or nonlinear relationships, while also improving robustness by penalizing outliers less severely than the product approach. Within the cell, the concentration of a specific metabolite is highly correlated with the enzymes responsible for its synthesis and utilization. Concurrently, for intercellular communication, these metabolites are relayed outside the cell through specific transport proteins. Given this, extracellular metabolite concentrations are intrinsically linked to their related enzymes and transport proteins. This prompted us to estimate the extracellular concentration of particular metabolites using the expression levels of enzymes and transport proteins, providing insights into intercellular communication via ML-R interactions. The specific steps of the pipeline are as follows:

1. Estimating extracellular metabolite concentration
  1 $$M=\sqrt[2]{{E}^{2}+{T}^{2}}$$

where *M* is the extracellular metabolite concentration, and *E* is the expression of the enzymes, determined as:

2 $$E=\sqrt[m]{{\prod }_{i=1}^{m}{P}_{i}}-\sqrt[n]{{\prod }_{j=1}^{n}{S}_{j}}$$

where *P* is the average expression of the enzymes that produces the metabolite, and *m* represents the number of the enzymes that produces the metabolite.* S* is the average expression of the enzyme that consumes the metabolite. *n* represents the number of enzymes that consumes the metabolite. And *T* is the expression of the transporter proteins for the metabolite. The calculation of *T* is formulated as follows:

3 $$T=\sqrt[k]{{\prod }_{g=1}^{k}{T}_{g}}$$

where *T*_*g*_ is the average expression of the transporter proteins, and *k* represents the number of transporter proteins associated with the metabolite. *g* is the *g*^th^ transporter proteins in the *k* transporter proteins.

2) Estimating receptor expression

Receptors, often a protein or complexes, bind or receive metabolites to trigger signaling. If a receptor is a complex containing *q* proteins, *R* is defined as the geometric mean of the expression value of all proteins. The formula is as follows:

4 $$R=\sqrt[q]{{\prod }_{h=1}^{q}{R}_{h}}$$

where *R*_*h*_ is the expression value of protein *h* in the receptor complex.

3) Inferring the cell-cell communication score

To infer the cell communication between different cell types based on metabolites and their receptor proteins. The *MR*_*score*_ is defined as the score of an ML-R interaction entry between different cell types, which is evaluated by the expression of metabolite and receptor. The formula is as follows:

5 $$MR_{score} = \sqrt[2]{M^2 + R^2}$$

where *M* is the extracellular concentration of metabolite, and *R* is the expression of receptor, respectively.

4) Estimating statistical significance of CCC score

To examine the statistical significance of each ML-R interaction entry, the *p*-value was estimated using the permutation test (by randomly permutating group labels of cells and then recalculating the communication strength for each permutation).

### Architecture

The MRCLinkdb runs on CentOS Linux. The architecture of the MRCLinkdb is composed of two parts. The first part is the frontend, which provides a visual interface of the webpage and an interactive experience with users by Hyper Text Markup Language 5 (HTML5), Cascading Style Sheets (CSS) and JavaScript. The last part is the backend. All data are stored in MySQL. PHP plays a key role in connecting the frontend and backend. PHP can execute different scripts in response to the user’s various requests. To streamline the development process and enhance functionality, various plugins were incorporated: DataTables, an addition to the jQuery JavaScript library, this plugin augments HTML tables with advanced features; ECharts, a declarative framework designed for swift creation of web-based visualizations; Bootstrap, a robust frontend toolkit that provides numerous features; Smarty, facilitates the separation of frontend and backend, isolating logical programs from external content. This ensures ease in subsequent management and maintenance.

## Data Availability

All data generated or analyzed during this study are included in this published article, its supplementary information files, and publicly available repositories. MRCLinkdb is free available at https://www.cellknowledge.com.cn/mrclinkdb/ to all users without any login or registration restrictions. We also integrated all the protein L-R interaction data (includes over 7400 human and 5800 mouse protein L-R interactions) from various platforms and share it on GitHub for free (link: https://github.com/ZhangCellab/Protein-Ligand-Receptor-Interactions). The data and code used for the study have been uploaded to Zenodo (DOI: 12602862, https://zenodo.org/records/12602862). Moreover, the codes for predicting intercellular metabolite-ligand-receptor interactions are stored on GitHub (https://github.com/ZhangCellab/MRCLinkdb) and are also available for free on MRCLinkdb (https://www.cellknowledge.com.cn/mrclinkdb/Download.html). Human and mouse single cell atlas were collected from Mouse Cell Atlas (MCA) version 1.0 and version 1.1. The bulk RNA-seq/microarray data for gene expression across different tissues in human and mouse were collected from the Human Protein Atlas (HPA) project and the TISSUES 2.0 database, respectively.

## Abbreviations

MRCLinkdb: Metabolite-Receptor based Cell Link Database
ML-R: Metabolite-ligand-receptor
L-R: Ligand-receptor
CCC: Cell-cell communication
PPIs: Protein-protein interactions
